# Performance of ChatGPT Across Different Versions in Medical Licensing Examinations Worldwide: Systematic Review and Meta-Analysis

**DOI:** 10.2196/60807

**Published:** 2024-07-25

**Authors:** Mingxin Liu, Tsuyoshi Okuhara, XinYi Chang, Ritsuko Shirabe, Yuriko Nishiie, Hiroko Okada, Takahiro Kiuchi

**Affiliations:** 1 Department of Health Communication Graduate School of Medicine The University of Tokyo Tokyo Japan; 2 Department of Health Communication School of Public Health Graduate School of Medicine, The University of Tokyo Tokyo Japan; 3 Department of Industrial Engineering and Economics School of Engineering Tokyo Institute of Technology Tokyo Japan

**Keywords:** large language model, ChatGPT, medical licensing examination, medical education, LLMs, NLP, natural language processing, artificial intelligence, language models, review methods, systematic, meta-analysis

## Abstract

**Background:**

Over the past 2 years, researchers have used various medical licensing examinations to test whether ChatGPT (OpenAI) possesses accurate medical knowledge. The performance of each version of ChatGPT on the medical licensing examination in multiple environments showed remarkable differences. At this stage, there is still a lack of a comprehensive understanding of the variability in ChatGPT’s performance on different medical licensing examinations.

**Objective:**

In this study, we reviewed all studies on ChatGPT performance in medical licensing examinations up to March 2024. This review aims to contribute to the evolving discourse on artificial intelligence (AI) in medical education by providing a comprehensive analysis of the performance of ChatGPT in various environments. The insights gained from this systematic review will guide educators, policymakers, and technical experts to effectively and judiciously use AI in medical education.

**Methods:**

We searched the literature published between January 1, 2022, and March 29, 2024, by searching query strings in Web of Science, PubMed, and Scopus. Two authors screened the literature according to the inclusion and exclusion criteria, extracted data, and independently assessed the quality of the literature concerning Quality Assessment of Diagnostic Accuracy Studies-2. We conducted both qualitative and quantitative analyses.

**Results:**

A total of 45 studies on the performance of different versions of ChatGPT in medical licensing examinations were included in this study. GPT-4 achieved an overall accuracy rate of 81% (95% CI 78-84; *P*<.01), significantly surpassing the 58% (95% CI 53-63; *P*<.01) accuracy rate of GPT-3.5. GPT-4 passed the medical examinations in 26 of 29 cases, outperforming the average scores of medical students in 13 of 17 cases. Translating the examination questions into English improved GPT-3.5’s performance but did not affect GPT-4. GPT-3.5 showed no difference in performance between examinations from English-speaking and non–English-speaking countries (*P*=.72), but GPT-4 performed better on examinations from English-speaking countries significantly (*P*=.02). Any type of prompt could significantly improve GPT-3.5’s (*P*=.03) and GPT-4’s (*P*<.01) performance. GPT-3.5 performed better on short-text questions than on long-text questions. The difficulty of the questions affected the performance of GPT-3.5 and GPT-4. In image-based multiple-choice questions (MCQs), ChatGPT’s accuracy rate ranges from 13.1% to 100%. ChatGPT performed significantly worse on open-ended questions than on MCQs.

**Conclusions:**

GPT-4 demonstrates considerable potential for future use in medical education. However, due to its insufficient accuracy, inconsistent performance, and the challenges posed by differing medical policies and knowledge across countries, GPT-4 is not yet suitable for use in medical education.

**Trial Registration:**

PROSPERO CRD42024506687; https://www.crd.york.ac.uk/prospero/display_record.php?RecordID=506687

## Introduction

### Background

In November 2022, the web-based artificial intelligence (AI) chatbot ChatGPT (OpenAI) was released to the public and swiftly garnered global attention because of its ability to provide detailed answers to complex queries [1]. ChatGPT has been extensively applied across various domains, including programming, education, business, and law, with notable success in each [2-5]. Researchers have been actively exploring the potential roles and capabilities of ChatGPT in clinical diagnosis, health care, and medical education [6,7]. The number of publications on this topic has increased dramatically since late 2022 [8,9]. Specifically, in medical education, ChatGPT can play several important roles, including, but not limited to, the following: First, compared to search engines like Google, which present a list of relevant pages, ChatGPT aims to provide concise and practical answers to users’ questions, making it an effective knowledge resource [10,11]. Second, in medical licensing examinations comprising multiple-choice questions (MCQs), ChatGPT can act as an “AI teaching assistant,” providing insights for each question, analyzing common errors, and reinforcing concepts interactively [12]. Third, ChatGPT has the capability to analyze images. Although this feature is still in its early stages, it offers the potential for ChatGPT to serve as a “virtual mentor,” capable of analyzing medical images such as skin rashes and x-rays [10]. Fourth, for most medical students who find it challenging to balance studying vast amounts of information, practicing evidence-based medicine, and fulfilling clinical duties, ChatGPT can provide concise summaries of clinical trials and generate key practical points from them [10].

However, a prerequisite for ChatGPT’s ability to help medical students in their studies and play a role in medical education, both now and in the future, is that ChatGPT has solid and accurate knowledge of medicine. Medical licensing examinations are a crucial part of the medical education pathway as they assess the readiness of aspiring doctors to enter clinical practice. These examinations vary in format and content across countries but typically test medical knowledge, clinical reasoning, and ethical decision-making [13]. Over the past 2 years, researchers have used medical licensing examinations from various countries to test whether ChatGPT possesses accurate medical knowledge [14-57].

Although most of these studies used similar testing methods—inputting medical licensing examination questions into ChatGPT and recording the responses to calculate accuracy—the ChatGPT performance showed significant variation. A study conducted in the United States revealed that GPT-3.5 surpassed the 60% score threshold on the National Board of Medical Examiners (NBME)-free–Step-1 question, reaching the level of a third-year medical student [21]. However, studies from South Korea, China, and Japan have indicated that GPT-3.5 failed to pass medical examinations in their respective countries [26,43,44,47,48,51,54]. Although GPT-4 performed better overall than GPT-3.5 [33,36,41,44,47], it did not pass the Japanese medical licensing examination [49]. In addition, ChatGPT performance varies significantly across medical specialties within these examinations [23,25-27,30,33-35].

At this stage, there is still a lack of a comprehensive understanding of the variability in ChatGPT’s performance on different medical licensing examinations. We believe that prematurely using ChatGPT for clinical diagnosis and medical education without thoroughly evaluating its performance across various medical licensing examinations is irresponsible and could endanger human lives.

### Literature Review

A total of 3 systematic reviews have explored ChatGPT’s performance in medical licensing examinations to the best of our knowledge [58-60].

A study from the United States collected literature up to June 2, 2023, focusing on various types of medical licensing examinations in the United States [58]. Among the 19 included studies, only 2 were comprehensive medical licensing examinations, the United States Medical Licensing Examination (USMLE), while the remaining 17 were medical specialty examinations, such as plastic surgery, anesthesia, and ophthalmology [58]. In contrast to this study, our research extends the literature collection to a global scale and examines the performance of ChatGPT in medical licensing examinations in different countries and languages. We believe that the worldwide perspective of the current review is crucial because medical education and licensure standards vary significantly across countries.

A study from Pakistan collected literature up to April 2023, focusing on the performance of GPT-3.5 in various medical licensing examinations worldwide [59]. However, with the advent of the more advanced GPT-4, more studies have focused on GPT-4. Our research includes all ChatGPT versions and discusses their performance differences.

A study from China collected the literature up to July 15, 2023 [60]. This study reviewed the performance of ChatGPT for various medical questions. Of the 60 included studies, only 3 were medical licensing examinations. In addition, this study created a framework to evaluate the quality of studies on the performance of large language models (LLMs) in medical questions [60]. We slightly modified this evaluation framework and applied it to this study.

### Study Aims and Objectives

This study reviewed all studies on ChatGPT’s performance in medical licensing examinations from January 1, 2022, to March 29, 2024, to clarify the following issues:

1. Can ChatGPT pass the medical licensing examinations?
2. How does ChatGPT’s performance compare to that of medical students?
3. How did ChatGPT perform in different languages?
4. What is the relationship between question difficulty and ChatGPT’s performance?
5. What is the relationship between question length and ChatGPT’s performance?
6. How did ChatGPT perform on image-based MCQ?
7. How did ChatGPT perform on open-ended questions?
8. What is the difference in ChatGPT’s performance with and without prompts?
9. Comparison of GPT-3.5’s and GPT-4’s performances.
10. How does ChatGPT perform in medical licensing examinations in English-speaking countries and non–English-speaking countries?

By comprehensively evaluating the accuracy of the medical knowledge held by ChatGPT, we integrate these perspectives and offer comprehensive recommendations for applying ChatGPT in medical education.

Overall, this systematic review aimed to fill the knowledge gap regarding the application of ChatGPT in medical licensing examinations. Further, it sought to contribute to the evolving discourse on AI in medical education and facilitate future developments and applications in this field. The insights gained from this systematic review will guide educators, policymakers, and technical experts to effectively and judiciously use AI in medical education.

To the best of our knowledge, this is the first study to comprehensively review the performance of all versions of ChatGPT on medical licensing examinations across different countries.

## Methods

This systematic review followed the PRISMA (Preferred Reporting Items for Systematic Reviews and Meta-Analyses) flow diagrams and guidance [61]. This systematic review was registered in the PROSPERO (International Prospective Register of Systematic Reviews) database on February 1, 2024 (CRD42024506687).

### Search Strategy

We searched for specific query strings (Multimedia Appendix 1) using the advanced search function in PubMed, Web of Science, and Scopus, with Google Scholar as a supplementary source. Literature published from January 1, 2022, to March 29, 2024, was included in the literature search. The literatures exported from these 3 platforms were imported into Rayyan [62]. Two authors (ML and XC) independently screened the titles and abstracts of the retrieved studies using a search strategy to identify those that met the inclusion and exclusion criteria (Textbox 1). The full texts of these studies were then retrieved and independently assessed for eligibility by 2 authors. Any disagreements regarding the eligibility of specific studies were discussed and resolved by a third reviewer (TO). In addition to the database searches, we searched Google Scholar for triangulations on March 29, 2024. When the preprint and peer-reviewed literature data were identical, we included the peer-reviewed literature in our analysis. As part of the screening process, we recorded the reasons for study exclusion and presented them in a prismatic flow diagram.

**Inclusion and exclusion criteria.**

**Inclusion criteria**

- The study tested the performance of ChatGPT in medical licensing examinations.
- Any type of original research literature (peer-reviewed papers, conference papers, preprints, letters, books, etc).
- Literature published from 2022 to 2024.
- Literature on the performance of ChatGPT in all languages.
- Literature on any version of ChatGPT.
- Literature on multiple-choice questions, open-ended questions, and all other types of questions for medical licensing examinations.

**Exclusion criteria**

- Nonnational-level medical licensing examination.
- Examinations other than comprehensive medical licensing examinations (eg, medical final examinations at universities, medical questions created by the authors themselves, and medical specialty examinations).
- Studies that are not related to ChatGPT.
- Duplicate studies.
- Studies that are not published in English.
- Systematic review.

### Data Extraction and Management

Two reviewers (ML and XC) independently extracted data from the included studies into an Excel (Microsoft Corp) spreadsheet by 2 reviewers (ML and XC). The data were compared, and inconsistencies were resolved through consensus or by a third reviewer (TO). The general characteristics to be extracted include the following: (1) title, (2) authors, (3) publication year, (4) publication date, (5) type of publication, (6) country of the medical licensing examination, (7) name of the medical licensing examination, (8) ChatGPT version, (9) language in which ChatGPT was tested, (10) duration of the test, (11) type of questions, (12) counts of correct or total questions, (13) accuracy rate, (14) did ChatGPT pass the examination, (15) comparison between medical students, and (16) was a prompt used.

### Assessing the Risk of Bias in the Included Studies

A previous study developed an LLM evaluation framework based on the Quality Assessment of Diagnostic Accuracy Studies-2 (QUADAS-2) [60,63]. We modified and applied this evaluation framework in our study (Multimedia Appendix 2).

Since this previous study collected papers on ChatGPT’s performance across all types of medical questions [60], we modified the original framework, whereas our research focused on ChatGPT’s performance in medical licensing examinations. Specifically, we added 2 evaluation items, items 4 and 5, to address aspects specific to medical licensing examinations. We removed item 8 (are the questions individual stand-alone queries or a continuous conversation requiring multiple consecutive inquiries?) from the original evaluation framework, as it did not apply to this study.

In our modified evaluation framework, “task generation,” “conversation structure,” and “evaluation” correspond to “patient selection,” “index test,” and “reference standard” in QUADAS-2, respectively. Items 2 and 7 correspond to “flow and timing” in QUADAS-2.

### Evidence Synthesis

Our analysis focuses on GPT-3.5 and GPT-4.

### Qualitative Analyses

We performed a comprehensive summary using narrative analysis and descriptive statistics for the contents of the included studies that were narrative or lacked sufficient data.

### Quantitative Analyses

We used the raw correct and total data in each included study to calculate the accuracy rate. The calculation rules are as follows: if a study used 1 set of questions for repeated testing, the displayed accuracy rate is the average score of all attempts and the total number of questions in the set. If the study tested both the original language and translated English questions, the displayed accuracy rate was based on the scores from the original language examination questions. For studies tested with and without optimized prompts, the displayed accuracy rate was based on the scores without optimized prompts. In studies that included MCQs and open-ended questions, the displayed accuracy rate excluded scores from the open-ended questions.

We conducted a meta-analysis of studies that tested ChatGPT using MCQs.

The *I*² statistic was used to assess the effect of heterogeneity on the pooled results. When significant heterogeneity was present (*I*²>50%), a random effects model was used; otherwise, a fixed effects model was used. Accuracy was reported with a 95% CI. The significance level was set at *P*<.05. Meta-regression and subgroup analyses were conducted to examine the potential sources of heterogeneity and compare performances across different subgroups. A sensitivity analysis was conducted to assess the robustness of the meta-analysis results. Accuracy was reported with 95% CIs. The “metafor” and “meta” packages in R (version 4.4.0; R Core Team) were used for the meta-analysis, publication bias, and sensitivity analyses.

In addition, we conducted post hoc power analysis for the random effects model results of each main group and subgroup. G*Power (version 3.1.9.7; Erdfelder, Faul, and Buchner) was used for the power analysis.

## Results

### Literature Screening and Selection

By searching the query strings in the Web of Science, Scopus, and PubMed, we retrieved 3698 papers from the Web of Science, 6354 papers from Scopus, and 2587 papers from PubMed. After excluding 3751 duplicate papers, 8888 papers remained. We excluded 278 non-English papers, leaving 8610 papers. After reading the abstracts of all 8610 papers, we excluded 8,377 studies that were completely irrelevant to this review, leaving 233 studies remaining.

A total of 137 focused on ChatGPT’s performance in medical specialty examinations, 11 on dental licensing examinations, 6 on nursing examinations, 6 on pharmacist examinations, and 25 on other medical examinations (eg, university medical entrance examinations and university medical final examinations). Further, 2 were systematic reviews, 1 was about nonnational medical examinations, and 2 lacked the necessary information. These studies did not meet the inclusion criteria.

**Figure 1:**
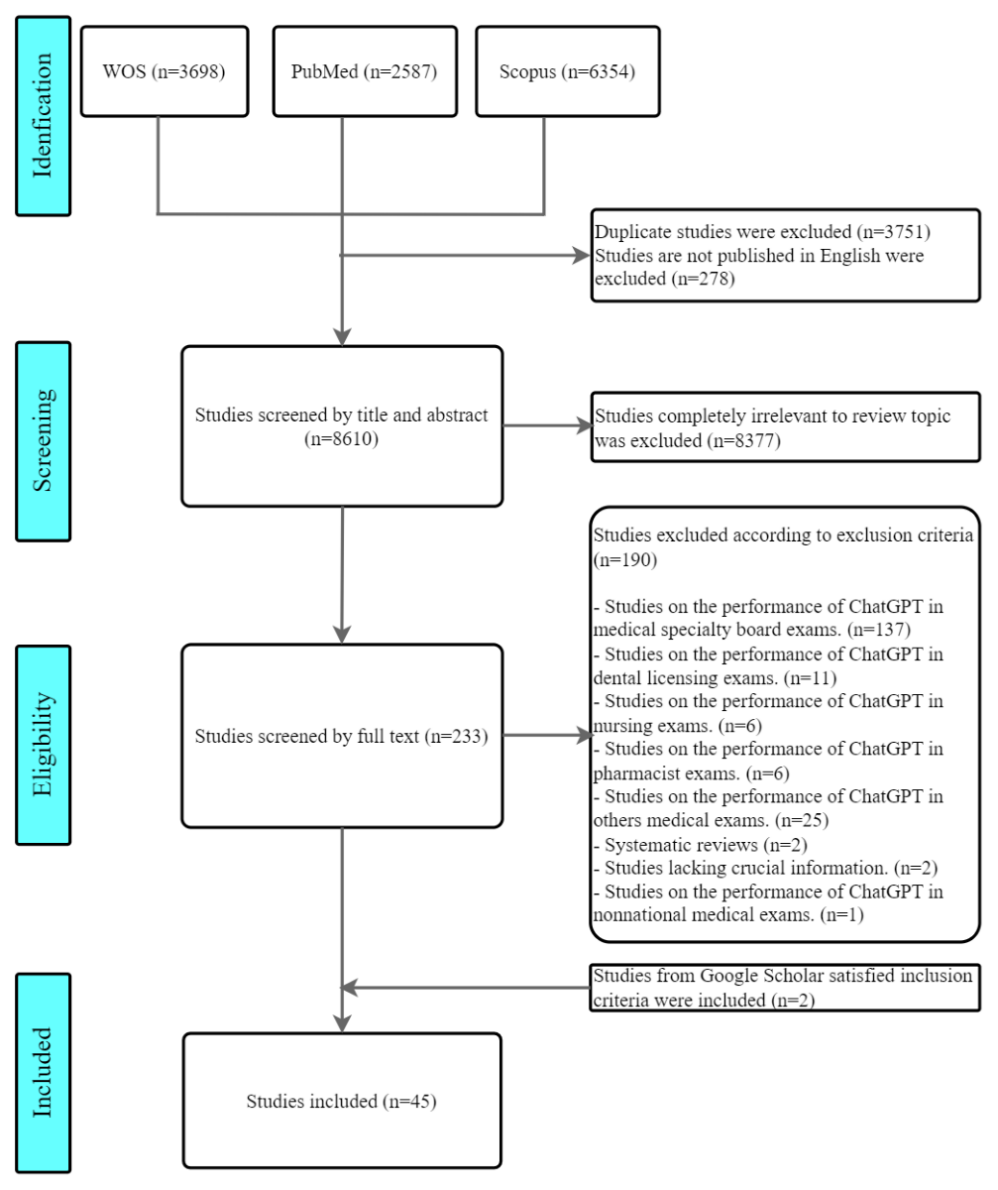
PRISMA (Preferred Reporting Items for Systematic Reviews and Meta-Analyses) flow diagram.

We then performed a supplementary search using Google Scholar and added 2 preprint papers on March 29, 2024. Ultimately, 45 papers were included in this systematic review (Figure 1) [3,14-57].

### Quality Assessment of Included Studies

**Figure 2:**
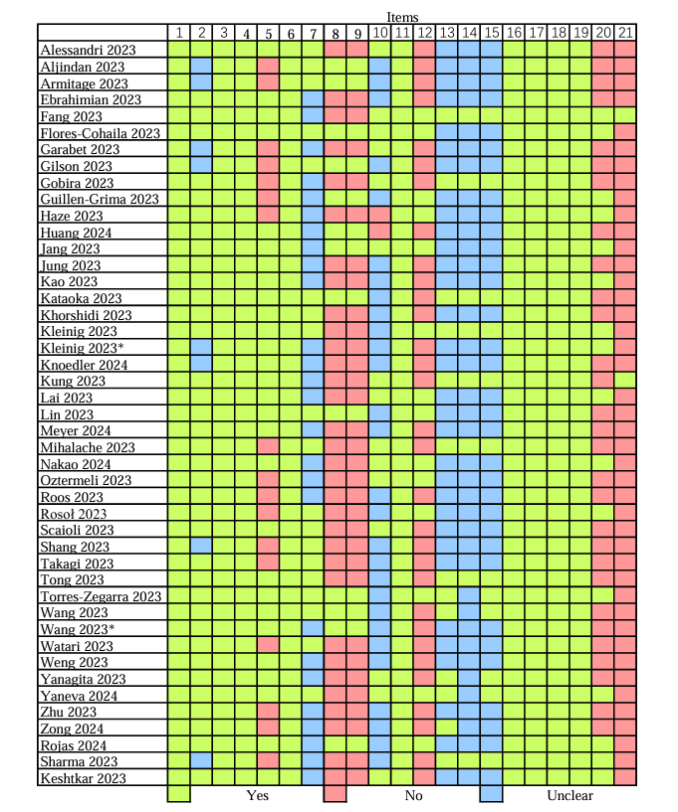
Quality assessment of included studies using evaluation framework.

Two authors independently assessed the quality of the 45 studies using an evaluation framework, and any disagreements were resolved through discussion and consensus (Figure 2). The literature we collected tested ChatGPT’s performance using national medical licensing examinations comprising MCQs with standard answers. Consequently, items 13, 14, 15, and 21 pertain to evaluators were not mentioned in three-quarters of the included studies. Unlike open-ended questions, MCQs do not require multiple evaluators to adopt a double-anonymized approach to evaluate test results. Therefore, this does not increase the risk in the “reference standard” part.

For item 7, more than half of the studies did not specify the exact test dates. On November 6, 2023, OpenAI developers announced that the cutoff dates for ChatGPT versions 3.5 and 4 were updated from September 2021 to January 2022 and April 2023, respectively [64]. We believe that if the cutoff date of ChatGPT is updated during the testing period, this might affect the consistency of ChatGPT’s performance before and after the update.

For item 10, more than half of the studies did not specify whether a new chat session was used to test different questions. Conducting different questions in the same session might have affected the ChatGPT performance.

For reasons above, in the risk of bias assessment, only 2 studies and 3 studies were rated as high risk in the “index test” and “flow and timing” categories, respectively (Table 1).

**Table 1:** Risk of bias.

Author (year) and reference	Patient selection	Index test	Reference standard	Flow and timing
Alessandri Bonetti et al (2024) [14]	Low	Low	Low	Low
Aljindan et al (2023) [15]	Unclear	Unclear	Low	Low
Armitage (2024) [16]	Unclear	Unclear	Low	Low
Ebrahimian et al (2023) [17]	Low	Unclear	Low	Unclear
Fang et al (2023) [18]	Low	Low	Low	Unclear
Flores-Cohaila et al (2023) [19]	Low	Low	Low	Low
Garabet et al (2023) [20]	Unclear	Low	Low	High
Gilson et al (2023) [21]	Unclear	Unclear	Low	Low
Gobira et al (2023) [22]	Low	Low	Low	Unclear
Guillen-Grima et al (2023) [23]	Low	Unclear	Low	Unclear
Haze et al (2023) [24]	Low	High	Low	Unclear
Huang et al (2024) [25]	Low	High	Low	Unclear
Jang et al (2023) [26]	Low	Low	Low	Unclear
Jung et al (2023) [27]	Low	Unclear	Low	Unclear
Kao et al (2024) [28]	Low	Unclear	Low	Unclear
Kataoka et al (2023) [29]	Low	Unclear	Low	Low
Khorshidi et al (2023) [30]	Low	Unclear	Low	Low
Kleinig et al (2023) [31]	Low	Unclear	Low	Low
Kleinig et al (2023) [32]	Low	Unclear	Low	High
Knoedler et al (2024) [33]	Low	Unclear	Low	Unclear
Kung et al (2023) [3]	Low	Low	Low	Unclear
Lai et al (2023) [34]	Low	Low	Low	Unclear
Lin et al (2024) [35]	Low	Unclear	Low	Low
Meyer et al (2024) [36]	Low	Unclear	Low	Unclear
Mihalache et al (2023) [37]	Low	Low	Low	Low
Nakao et al (2024) [38]	Low	Low	Low	Unclear
Oztermeli and Oztermeli (2023) [39]	Low	Low	Low	Unclear
Roos et al (2023) [40]	Low	Unclear	Low	Unclear
Rosoł et al (2023) [41]	Low	Unclear	Low	Low
Scaioli et al (2023) [42]	Low	Low	Low	Low
Shang et al (2023) [43]	Unclear	Unclear	Low	Low
Takagi et al (2023) [44]	Low	Unclear	Low	Low
Tong et al (2023) [45]	Low	Unclear	Low	Low
Torres-Zegarra et al (2023) [46]	Low	Unclear	Low	Low
Wang et al (2023) [47]	Low	Unclear	Low	Low
Wang et al (2023) [48]	Low	Unclear	Low	Unclear
Watari et al (2023) [49]	Low	Unclear	Low	Low
Weng et al (2023) [50]	Low	Unclear	Low	Unclear
Yanagita et al (2023) [51]	Low	Low	Low	Unclear
Yaneva et al (2024) [52]	Low	Low	Low	Low
Zhu et al (2023) [53]	Low	Unclear	Low	Unclear
Zong et al (2024) [54]	Low	Unclear	Low	Unclear
Rojas et al (2024) [55]	Low	Unclear	Low	Unclear
Kung et al (2023) [56]	Unclear	Unclear	Low	High
Keshtkar et al (2023) [57]	Low	Low	Low	Low

### General Characteristics of Included Studies

Among the 45 reviewed papers, the earliest was published on February 8, 2023 [21], and the latest on April 30, 2024 [55]. The general characteristics of the studies are shown in Multimedia Appendix 3.

**Figure 3:**
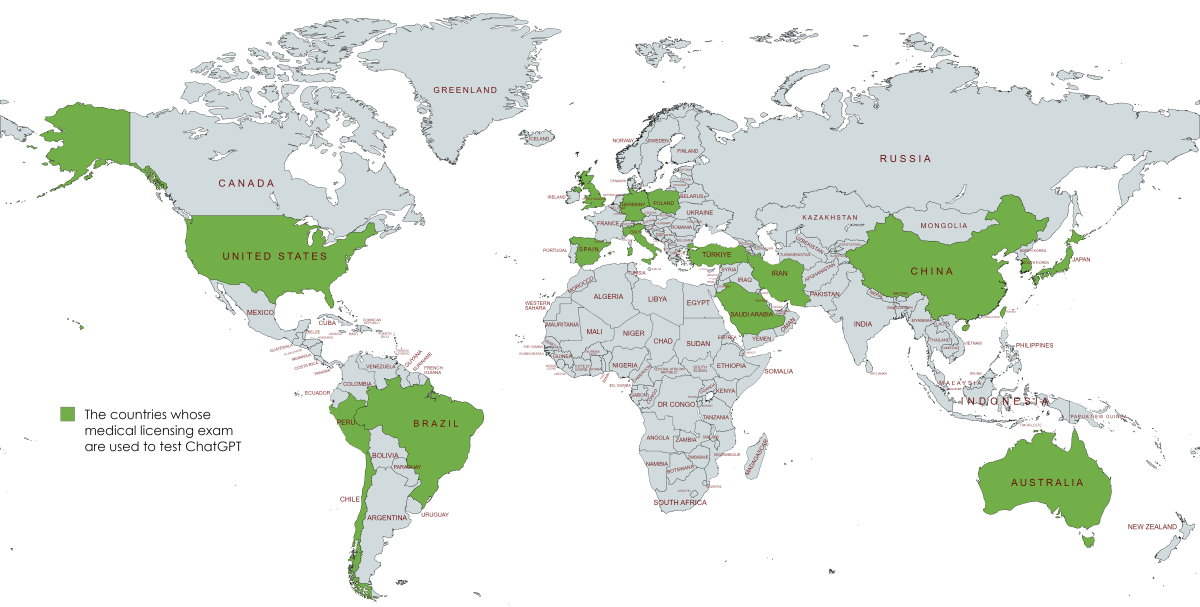
Countries where medical licensing examination was used to test ChatGPT.

The medical licensing examinations applied to test ChatGPT’s performance were from 17 countries and regions: Italy (n=2), Saudi Arabia (n=1), the United Kingdom (n=2), Iran (n=3), China (n=7), Peru (n=2), the United States (n=7), Brazil (n=1), Spain (n=1), Japan (n=6), Taiwan (n=4), South Korea (n=1), Germany (n=3), Australia (n=2), Turkey (n=1), Poland (n=1), and Chile (n=1; Figure 3).

Of the 45 included studies, 29 tested the performance of GPT-4, and 26 tested the performance of GPT-3.5. A total of 14 studies tested both GPT-4 and GPT-3.5. In addition, 4 studies tested the GPT-3, one tested the InstructGPT, and one tested the ChatGPT Plus.

Regarding the countries and languages of the medical licensing examination questions used to test ChatGPT, 11 studies used examinations from an English-speaking country. Of the 34 medical licensing examinations of non–English-speaking countries, 22 used only the native language for testing, three translated the original language into English, and 9 used both the original and translated English questions.

All 45 studies included MCQs, with 4 studies including open-ended questions, 1 study including calculation questions, and 1 study including patient history inquiry questions.

### Qualitative Analyses

**Figure 4:**
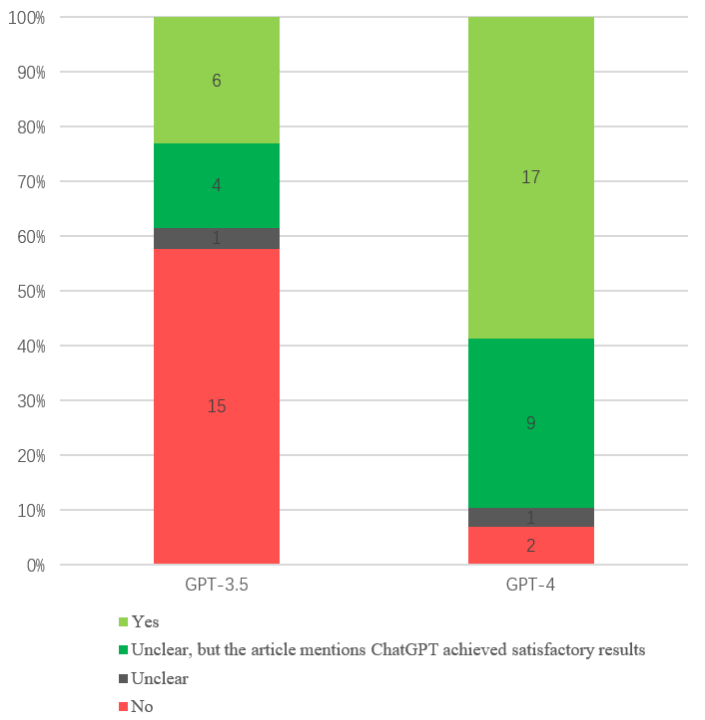
Performance of ChatGPT on passing the medical licensing examination.

Regarding the performance of ChatGPT on passing the medical licensing examination, among the 26 studies testing GPT-3.5, 6 reported that GPT-3.5 passed the medical licensing examination, and 4 reported satisfactory performance, making up 38.5% (10/26) of the total. In the remaining studies, 1 was unclear, and 15 did not pass. Among the 29 studies testing GPT-4, 17 reported that GPT-4 passed the medical licensing examination, and 9 reported satisfactory performance, making up 89.7% (26/29) of the total. In the remaining studies, 1 was unclear and 2 did not pass (Figure 4). For the other ChatGPT models, among the 4 studies testing the GPT-3 performance, 2 did not pass, 1 was unclear, and 1 showed a satisfactory performance. The studies that tested GPT-4 with Vision (GPT-4V, which is specifically designed for image tasks), InstructGPT, and ChatGPT Plus showed the following results: passed, did not pass, and did not pass.

**Figure 5:**
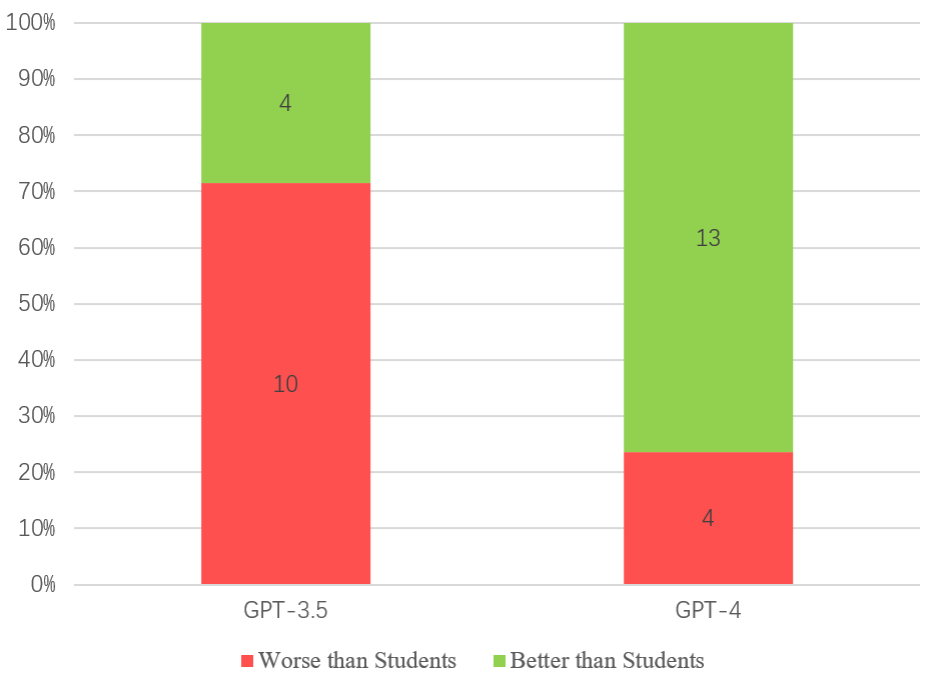
Performance of ChatGPT compared with medical students.

Regarding the performance of ChatGPT compared with medical students, 14 of 45 studies compared GPT-3.5’s performance with medical students, and 17 of 45 compared GPT-4’s performance with that of medical students. Four studies showed that GPT-3.5 surpassed medical students, accounting for 28.6% (4/14) of the studies. A total of 13 studies showed that GPT-4 surpassed medical students, accounting for 76.5% (13/17) of the studies (Figure 5). For the other ChatGPT models, 1 study showed that GPT-3 surpassed medical students, while another showed that it performed worse. One study indicated that InstructGPT performed worse than the students.

We also compared ChatGPT’s performance in the original language and English-translated questions of the same non-English medical licensing examination. In studies of medical licensing examinations in non–English-speaking countries, 9 used both the original language and English-translated questions to test ChatGPT’s performance, with 8 reporting comparative results (Table 2). Overall, for GPT-4, translating the original language into English had a limited effect on improving the performance. The accuracy improvement ranged from 0.17% to 8.65%, with 6 studies showing an accuracy increase of less than 5%. However, compared with GPT-4, GPT-3.5 showed significant improvement when tested in English in 4 studies. In 2 of these studies, GPT-3.5’s accuracy was more than 20% higher in English than in the original language.

**Table 2:** ChatGPT’s performance in original language and English-translated questions [18,23,26,30,41,45,47,57].

Author (year) and reference	GPT-3.5 accuracy rate		GPT-4 accuracy rate		
	Original language, n/n (%)	English-translated, n/n (%)	Original language, n/n (%)	English-translated, n/n (%)	
Fang et al (2023) [18]	Untested	Untested	197/260 (75.77%)	201/260 (77.31%)	
Guillen-Grima et al (2023) [23]	115/182 (63.2)	121/182 (66.5)	158/182 (86.8)	160/182 (87.9)	
Jang et al (2023) [26]	Untested	Untested	Unclear (51.8)	Unclear (60.5)	
Khorshidi et al (2023) [30]	Untested	Untested	161/198 (81.3)	167/198 (84.3)	
Rosoł at al (2023) [41]	320.5/585 (54.8)	353/585 (60.3)	465.5/585 (79.6)	466.5/585 (79.7)	
Tong at al (2023) [45]	Untested	Untested	130/160 (81.3)	138/160 (86.3)	
Wang at al (2023) [47]	56/100 (56)	76/100 (76)	84/100 (84)	86/100 (86)	
Keshtkar at al (2023) [57]	394/1105 (35.7)	687/1105 (61.4)	Untested		Untested

A total of 2 and 3 studies examined the correlation between GPT-3.5 or GPT-4 performance and the length of the question text, respectively. Both studies on GPT-3.5 showed a significant correlation between performance and the length of the question text; the longer the question text, the poorer the performance of GPT-3.5 [33,39]. In contrast, none of the 3 studies on GPT-4 found a significant difference in performance between long- and short-text questions [23,37,50].

A total of 8 studies examined the correlation between the difficulty of the questions and ChatGPT’s performance. A total of 7 studies indicated that both GPT-4 and GPT-3.5 performed worse on difficult questions than easier ones [21,23,30,33,41,44,49]. Only 1 study showed that the difficulty of the questions did not affect GPT-4’s performance. However, in this study, the difficulty was subjectively rated by 3 medical students rather than using official difficulty ratings [45].

Regarding ChatGPT’s performance with and without optimized prompts, in our review of 45 papers, 13 stated that researchers provided ChatGPT with prompts before asking questions. Most of these prompts were designed to help ChatGPT better understand its task, such as “You are now an experienced clinician; please answer the following questions” or “You are a medical student, and we will be using medical licensing examination questions to test you; please provide your best answers.” Researchers have not analyzed or elaborated on the impact of these task understanding prompts on ChatGPT’s performance. However, 3 studies used optimized prompts [19,26,35]. A Korean study used 4 kinds of optimized prompts, including annotating Chinese terms in traditional Korean medicine, translating the instruction and question into English, providing examination-optimized instructions, and using self-consistency in the prompt. The results showed that ChatGPT’s accuracy increased from 51.82% to 66.18% with optimized prompts [26]. In the other 2 studies, questions that ChatGPT initially answered incorrectly without prompts were reasked with optimized prompts, such as “Are you sure? Pretend to be a junior doctor with expertise in clinical practice and examination solving and retry” or “Could you double-check the answer?” ChatGPT could correctly answer up to 88.9% and 84% of these questions, respectively [19,35]. For task understanding prompts, we conducted a subgroup analysis and meta-regression to examine whether they affected ChatGPT’s performance.

Regarding the capability of ChatGPT in answering image-based MCQs, 4 studies have reported the performance of ChatGPT in image-based MCQs. Three tested GPT-4, and 1 compared GPT-4 and GPT-4V [16,23,38,55]. In a UK study, GPT-4 achieved an accuracy rate of 100% (3/3) for the image-based MCQs correctly [16]. In a Spanish study, the accuracy rate of GPT-4 for image-based MCQs in Spanish was 13%, and the accuracy rate was 26% after translating the image-based MCQs into English, twice as high as in Spanish [23]. Japanese researchers tested GPT-4’s performance on image-based MCQs that provided both images and text and on image-based MCQs that provided only text. The rate of correctness was 68% (73/108) when both images and text were provided, and 72% (78/108) when only text was provided [38]. Researchers in Chile compared the performance of ChatGPT-4 and ChatGPT-4V in image-based MCQs. The accuracy rates of GPT-4 and GPT-4V for image-based MCQs were 76.7% and 70%, respectively [55].

Regarding the performance of ChatGPT on questions other than MCQs, 4 studies compared ChatGPT’s performance on open-ended questions versus MCQs. Among them, 2 showed that ChatGPT performed significantly worse on open-ended questions than on MCQs [3,19], 1 showed slightly better performance on open-ended questions, and another asked ChatGPT 10 short questions, all of which received an “A” grade [28,56]. In a study using calculation questions from the Japanese medical licensing examination, ChatGPT’s performance on calculation questions was significantly worse than that of MCQs [24]. In a study using patient history inquiry questions from the Chinese medical licensing examination to assess medical students’ clinical skills, ChatGPT passed the test and scored higher than the average medical student, achieving satisfactory performance [53].

### Meta-Analysis

We conducted a meta-analysis of the integrated accuracy of GPT-3.5 and GPT-4 in medical licensing examinations. The accuracy of the meta-analysis was displayed in Multimedia Appendix 3. A total of 25 studies reporting the accuracy of GPT-3.5 and 29 studies reporting the accuracy of GPT-4 were included in this meta-analysis. Owing to significant heterogeneity (GPT-3.5: *I*²=95% and GPT-4 *I*²=93%), both groups were analyzed using a random-effects model.

The integrated accuracy for GPT-3.5 was 58% (95% CI 53-63; *P*<.01), and the integrated accuracy for GPT-4 was 81% (95% CI 78-84; *P*<.01; Figures S1 and S2 in Multimedia Appendix 4).

### Meta-Regression and Subgroup Analysis

We divided studies with GPT-3.5 and GPT-4 in Figures S1 and S2 in Multimedia Appendix 4 into 3 subgroups, respectively.

Subgroup 1 divided the studies into those using medical licensing examinations from English-speaking countries to test ChatGPT and those using examinations from non–English-speaking countries with a native language. Subgroup 2 categorized studies based on whether they used prompts to test ChatGPT or not. In Figures S3 and S4 in Multimedia Appendix 4, “yes” indicates the use of prompts, while “no” indicates the absence of prompts. Subgroup 3 categorized studies according to the “flow and timing” evaluation in Table 1, with “low risk” forming 1 category and “unclear” and “high risk” forming another. In Figures S5 and S6 in Multimedia Appendix 4, “yes” means “low risk,” implying that ChatGPT’s performance might not be affected by the testing date and source date. “No” means “high risk” and “unclear,” implying that ChatGPT’s performance might be influenced by the testing date and source date. We conducted meta-regression and subgroup analyses for all subgroups to examine potential sources of heterogeneity and compare performances.

In subgroup analysis of subgroup 1, because of significant heterogeneity (GPT-3.5 tested in medical licensing examinations of English-speaking countries: *I*²=80%, GPT-3.5 tested in original language examinations of non–English-speaking countries: *I*²=96%, GPT-4 tested in medical licensing examinations of English-speaking countries: *I*²=69%, and GPT-4 tested in original language examinations of non–English-speaking countries: *I*²=93%), all 4 groups were analyzed using a random-effects model.

The integrated accuracy for GPT-3.5 in examinations from English-speaking countries was 57% (95% CI 52-62; *P*<.01), and in examinations from non–English-speaking countries with original languages, it was 58% (95% CI 52-64; *P*<.01). No statistically significant differences were observed (*P*=.72; Figure S7 in Multimedia Appendix 4).

For GPT-4, the integrated accuracy in examinations from English-speaking countries was 86% (95% CI 82%-89%; *P*<.01), and in examinations from non–English-speaking countries with original languages, it was 80% (95% CI 76-83; *P*<.01). Statistically significant differences were observed between the results (*P*=.02; Figure S8 in Multimedia Appendix 4).

In the subgroup analysis of subgroup 2, because of significant heterogeneity (GPT-3.5 in subgroup “yes:” *I*²=92%, GPT-3.5 in subgroup “no:” *I*²=95%, GPT-4 in subgroup “yes:” *I*²=68%, and GPT-4 in subgroup “no:” *I*²=94%), all 4 groups were analyzed using a random-effects model.

The integrated accuracy for GPT-3.5 in examinations with prompts was 68% (95% CI 57-77; *P*<.01), and in examinations without prompts, it was 54% (95% CI 50-59; *P*<.01). Statistically significant differences were observed between the results (*P*=.03; Figure S3 in Multimedia Appendix 4).

The integrated accuracy for GPT-4 in examinations with prompts was 85% (95% CI 83-88; *P*<.01), and in examinations without prompts, it was 79% (95% CI 75-82; *P*<.01). Statistically significant differences were observed between the results (*P*<.01; Figure S4 in Multimedia Appendix 4).

In the subgroup analysis of subgroup 3, because of significant heterogeneity (GPT-3.5 in subgroup “yes:” *I*²=96%, GPT-3.5 in subgroup “no:” *I*²=92%, GPT-4 in subgroup “yes:” *I*²=71%, and GPT-4 in subgroup “no:” *I*²=95%), all 4 groups were analyzed using a random-effects model.

The integrated accuracy for studies in which GPT-3.5’s performance may be influenced by testing date and source date was 55% (95% CI 51-60; *P*<.01), and in studies in which GPT-3.5’s performance may not be influenced, it was 62% (95% CI 53-71; *P*<.01). No statistically significant differences were observed (*P*=.19; Figure S5 in Multimedia Appendix 4).

The integrated accuracy for studies in which GPT-4’s performance may be influenced by testing date and source date was 80% (95% CI 75-83; *P*<.01), and in studies in which GPT-4’s performance may not be influenced, it was 83% (95% CI 80-86; *P*<.01). No statistically significant differences were observed (*P*=.12; Figure S6 in Multimedia Appendix 4).

Regarding the meta-regression results for all subgroups, the use of prompts is likely to be a source of potential heterogeneity and showed a significant effect on the accuracy rates of GPT-3.5 and GPT-4 (subgroup 2), as indicated by an estimated regression coefficient of 0.54 (*P*=.01) and 0.46 (*P*=.02), respectively. Meta-regression of subgroups 1 and 3 did not show statistically significant effects on accuracy rates (all *P*>.05; Table 3).

**Table 3:** Meta-regression results of 3 subgroups of GPT-3.5 and GPT-4.

Version			Estimated regression coefficient		*P* value
**GPT-3.5**					
	Subgroup 1	–0.03		.91	
	Subgroup 2	0.54		.01	
	Subgroup 3	0.28		.19	
**GPT-4**					
	Subgroup 1	–0.39		.08	
	Subgroup 2	0.46		.02	
	Subgroup 3	0.25		.18	

### Publication Bias

No publication bias was detected among the included studies, as indicated by the funnel plots (Figure S9 in Multimedia Appendix 4).

### Sensitivity Analyses

We used a random effects model to assess the impact of excluding individual studies on overall effects. The sensitivity analysis plot showed that no single study significantly affected the overall meta-analysis results. This demonstrates the robustness of the meta-analysis results (Figure S10 in Multimedia Appendix 4).

### Power Analysis

We conducted post hoc power analysis for the main groups and subgroups using the results of the random effects model (Table 4). Subgroup 1 of GPT-3.5 had a power of 0.17. In this subgroup, we believe the sample size is adequate. The low power might be due to 2 main reasons. First, the intergroup difference is minimal, with effect sizes being very close (58% and 57%). Second, the data may have high heterogeneity (*I*²=80% and 96%). In the main group and other subgroups, the power was 1 or close to 1, indicating sufficient power to detect the anticipated effect size with the given sample size for the random effects model.

**Table 4:** Power analysis results of main groups and subgroups.

Versions and groups		Power
GPT-3.5	Main group (integrated accuracy rate in Figure S1 in Multimedia Appendix 4)	1
GPT-4	Main group (integrated accuracy rate in Figure S2 in Multimedia Appendix 4)	1
**GPT-3.5**		
	Subgroup 1	0.17
	Subgroup 2	1
	Subgroup 3	1
**GPT-4**		
	Subgroup 1	1
	Subgroup 2	1
	Subgroup 3	0.98

## Discussion

### Principal Findings

Our systematic review and meta-analysis are the first to comprehensively evaluate the performance of all versions of ChatGPT across various medical licensing examination environments. Overall, GPT-4 significantly outperformed GPT-3.5; however, there are still some issues that make it difficult to use in medical education at this stage.

Regarding the accuracy of ChatGPT on MCQs, while 2 previous studies conducted meta-analyses that yielded accuracy rates of 61% and 56%, respectively, we noted that these accuracy rates reflected the performance of all versions of ChatGPT without differentiation by version [54,56]. Our review found that GPT-4 achieved an integrated accuracy rate of 81% for MCQs in medical licensing examinations, passing nearly all tested examinations and surpassing the average performance of medical students in three-quarters of the tests. In contrast, GPT-3.5 achieved an integrated accuracy rate of 58%, failing to pass more than half of the medical examinations and surpassing the average performance of medical students in only 4 of 14 tests. Therefore, regarding accuracy rate, passing rate, and comparison with medical students, GPT-4 significantly surpassed GPT-3.5.

In medical licensing examinations from non–English-speaking countries, translating the original language questions into English significantly improved GPT-3.5’s performance but did not affect GPT-4’s performance. This indicates that GPT-4 has a much higher proficiency in languages other than English than GPT-3.5. However, based on the results of subgroup analysis for comparing GPT-3.5 and GPT-4 in medical licensing examinations from English-speaking and non–English-speaking countries, we found that GPT-4 performed better in English-speaking countries. In contrast, GPT-3.5 showed no performance difference between examinations from English-speaking and non–English-speaking countries.

Additionally, based on the results of qualitative analysis and subgroup analysis, we found that both “optimized prompts” and “task understanding prompts” could significantly improve ChatGPT’s performance. When using prompts, the accuracy rates of GPT-3.5 and GPT-4 were 68% and 85%, respectively, which were significantly higher than the accuracy rates of 54% and 79% without prompts.

The testing date and source date of each study were not sources of potential heterogeneity and did not significantly affect the performance of ChatGPT.

### Challenge of Using ChatGPT in Medical Education

First, although the AI hallucinations of GPT-4 have significantly been reduced compared to earlier versions, GPT-4 still generates incorrect information because the data used to train these models is not always correct [65]. We observed that in all tests of GPT-4, only 2 instances achieved an accuracy rate above 90%. The only example of a perfect accuracy rate was in the UK study, in which GPT-4 correctly answered all 20 questions [16]. However, the number of questions used in this test was significantly lower than those used in other studies. We believe that this demonstrates ChatGPT’s potential for future use in medical education but does not imply that medical students can rely on ChatGPT to acquire medical knowledge or prepare for examinations. Traditional sources of medical knowledge, such as medical school courses and textbooks, are completely reliable. However, because most professional medical knowledge exists in book form [50] and medical expertise on the internet is not always reliable [66], the medical knowledge that ChatGPT currently holds is not entirely accurate. In this context, if medical students rely on ChatGPT as a trusted source of expertise and acquire incorrect medical knowledge, the reliability of their knowledge and skills is significantly compromised. This is unacceptable in the medical field, as it directly impacts human lives. Therefore, GPT-4 passing medical licensing examinations does not imply that it can be used as a source of knowledge in medical education.

Previous studies have noted that the responses generated by GPT-3.5 are nondeterministic and random [67-69]. This study found that although the stability of GPT-4 has significantly improved compared to that of GPT-3.5, it still exhibits a degree of randomness in its outputs. Although GPT-4 achieved an overall accuracy of 81% across all tests, it only scored 52% on the Korean medical licensing examination, even lower than the overall accuracy of GPT-3.5 (58%) [25]. In addition, in 4 studies using Japanese medical licensing examination questions, although GPT-4 passed 3 of the tests, it only achieved an accuracy of 67% in one and did not pass [23,38,44,51]. Furthermore, the use of optimized prompts and the difficulty of the questions can affect ChatGPT’s performance stability. If millions of medical students use ChatGPT for learning, this randomness could be significantly magnified and affect their learning outcomes.

Moreover, different countries’ medical policies, and cultural, ethical, and unique local traditional medical knowledge pose significant challenges for ChatGPT [70]. Regarding varying medical policies and ethics, a Chinese study mentioned that abortion is prohibited in the United States but allowed in certain circumstances in China [48]. Although euthanasia is legal in many countries, it is illegal in Japan. ChatGPT chose the option of euthanasia in the Japanese medical licensing examination [25]. ChatGPT may struggle to adapt to localized medical policies and ethics. In addition, East Asian countries still use local traditional medicine (eg, Chinese medicine), and most local traditional medicine learning materials are written in the native languages. These materials might not be accessible on the internet and included in ChatGPT’s training data set, making it difficult for ChatGPT to provide accurate answers to such topics [18,26,50,54].

In the evaluation of image-based questions, we observed significant variations in the performance of GPT-4, with accuracy rates ranging from 13% to 100% [16,23,38,55]. However, there were only 3 questions in which GPT-4 achieved 100% accuracy, which is too small a sample size to demonstrate its proficiency in handling image-based questions [16]. In addition, a study from Japan tested the performance of ChatGPT when provided with images and text versus text only. Surprisingly, ChatGPT performed better when given only text than when provided with both images and text [38]. Similarly, Chile found that GPT-4V, designed explicitly for image tasks, performed worse on image-based questions than GPT-4 [55]. We believe that studies testing the ChatGPT’s performance on image-based questions are limited at this stage. Therefore, comprehensive and reliable conclusions cannot be drawn. Consequently, using ChatGPT for image-based medical education is extremely risky.

Finally, human teachers usually recognize their knowledge limitations when faced with uncertain questions and correct their mistakes by consulting resources. However, the fatal issue with ChatGPT is that, owing to the nature of AI language models, it can provide detailed and logically sound explanations for incorrect answers [24,40,44]. Given ChatGPT’s authoritative writing style, students are likely to believe and memorize the incorrect information provided by ChatGPT [71].

### Limitations

This systematic review did not include studies on the performance of ChatGPT in various medical specialty examinations, dental licensing examinations, pharmacy examinations, and other medical-related assessments. Future studies should review the performance of ChatGPT in these specific medical fields. Studies published in languages other than English were excluded from the systematic review. This may omit the literature that tests the performance of ChatGPT on non–English-speaking medical licensing examinations.

### Conclusions

A total of 45 studies on the performance of different versions of ChatGPT in medical licensing examinations were included in this systematic review. GPT-4 achieved an overall accuracy rate of 81%, significantly surpassing GPT-3.5, and, in most cases, passed the medical examinations, outperforming the average scores of medical students. Thus, GPT-4 demonstrates considerable potential for future use in medical education. However, because the knowledge of ChatGPT is not entirely accurate and its performance can be inconsistent, and because of the challenges posed by differing medical policies and knowledge across countries, we believe that GPT-4 is not yet suitable for use in medical education.

## Appendix

Multimedia Appendix 1 Query strings of Web of Science, Scopus, and PubMed.

Multimedia Appendix 2 Evaluation framework used in this systematic review.

Multimedia Appendix 3 General characteristics of included studies.

Multimedia Appendix 4 Supplementary figures.

Multimedia Appendix 5 PRISMA (Preferred Reporting Items for Systematic Reviews and Meta-Analyses) 2020 checklist.

## Abbreviations

AI: artificial intelligence
LLM: large language model
MCQ: multiple-choice question
NBME: National Board of Medical Examiners
PRISMA: Preferred Reporting Items for Systematic Reviews and Meta-Analyses
PROSPERO: International Prospective Register of Systematic Reviews
QUADAS-2: Quality Assessment of Diagnostic Accuracy Studies-2
USMLE: United States Medical Licensing Examination
