# Supplementary material for: Performance of ChatGPT Across Different Versions in Medical Licensing Examinations Worldwide: Systematic Review and Meta-Analysis
Source: J Med Internet Res. 2024 Jul 25;26:e60807. doi: 10.2196/60807 (PMC11310649; doi:10.2196/60807)
Supplement: Multimedia Appendix 1 [file jmir_v26i1e60807_app1.docx]

**Table S1**.

| Web of Science | TS = (("ChatGPT" OR "GPT" OR "Generative pre-trained transformer" OR "AI" OR "artificial intelligence" OR "chatbot*" OR "large language model*" OR "LLM") AND ("medical licensing exam*" OR "medical education" OR "medical license" OR "licensing" OR "license" OR "exam" OR "exams" OR "examination"))  OR  TI = (("ChatGPT" OR "GPT" OR "Generative pre-trained transformer" OR "AI" OR "artificial intelligence" OR "chatbot*" OR "large language model*" OR "LLM") AND ("medical licensing exam*" OR "medical education" OR "medical license" OR "licensing" OR "license" OR "exam" OR "exams" OR "examination"))  OR  AB = (("ChatGPT" OR "GPT" OR "Generative pre-trained transformer" OR "AI" OR "artificial intelligence" OR "chatbot*" OR "large language model*" OR "LLM") AND ("medical licensing exam*" OR "medical education" OR "medical license" OR "licensing" OR "license" OR "exam" OR "exams" OR "examination"))  OR  AK = (("ChatGPT" OR "GPT" OR "Generative pre-trained transformer" OR "AI" OR "artificial intelligence" OR "chatbot*" OR "large language model*" OR "LLM") AND ("medical licensing exam*" OR "medical education" OR "medical license" OR "licensing" OR "license" OR "exam" OR "exams" OR "examination")) |
| --- | --- |
| Scopus | TITLE-ABS-KEY (("ChatGPT" OR "GPT" OR "Generative pre-trained transformer" OR "AI" OR "artificial intelligence" OR "chatbot*" OR "large language model*" OR "LLM") AND ("medical licensing exam*" OR "medical education" OR "medical license" OR "licensing" OR "license" OR "exam" OR "exams" OR "examination")) |
| PubMed | ("ChatGPT" OR "GPT" OR "Generative pre-trained transformer" OR "AI" OR "artificial intelligence" OR "chatbot*" OR "large language model*" OR "LLM") AND ("medical licensing exam*" OR "medical education" OR "medical license" OR "licensing" OR "license" OR "exam" OR "exams" OR "examination") |
