# Supplementary material for: Performance of ChatGPT Across Different Versions in Medical Licensing Examinations Worldwide: Systematic Review and Meta-Analysis
Source: J Med Internet Res. 2024 Jul 25;26:e60807. doi: 10.2196/60807 (PMC11310649; doi:10.2196/60807)
Supplement: Multimedia Appendix 2 [file jmir_v26i1e60807_app2.docx]

**Table S1**.

| Section | Item | Checklist item |
| --- | --- | --- |
| Task Generation  (Patient Selection) | 1 | Source of the task (e.g., website, clinical vignette, exam question bank, book) |
|  | 2 | Source date (e.g., the question was sourced from the Medical Licensing Examination on February 28th, 2021) |
|  | 3 | Number (of the question/task) |
|  | 4 | Type of question |
|  | 5 | Did the study mention that ChatGPT does not know questions before it was tested. |
| LLM Version | 6 | LLM version (e.g., GPT-4) |
|  | 7 | Date of inquiry (e.g., the question was posed to ChatGPT on March 10th, 2023) |
| Conversation Structure  (Index Test) | 8 | Prompt of the inquiry (e.g., "Please answer…" or "Please role-play as a doctor and respond…") |
|  | 9 | Mode of inquiry: on-page or API call |
|  | 10 | Is the inquiry independent? (e.g., new chat) |
|  | 11 | Language (e.g., English, Chinese, Dutch) |
|  | 12 | Repetition of the inquiry: Is the inquiry repeated, and if so, how many repetitions? |
| Evaluation  (Reference Standard) | 13 | Who is the evaluator? (e.g., Internal medicine doctor, Pediatrician, Radiologist) |
|  | 14 | Is the expert assessor blinded? Is the expert assessor unaware of whether they are conversing with a human or an AI? |
|  | 15 | Number of evaluators |
|  | 16 | Evaluation metrics (e.g., Accuracy, Completeness, Safety, Empathy, Text length) |
|  | 17 | Does the response address the core question of the conversation |
|  | 18 | Overall response evaluation: the agreed evaluation metrics should be used to give an overall evaluation for the response |
|  | 19 | Is quantitative evaluation employed? If yes, what are the quantitative metrics? (e.g., Likert scale, Accuracy rate) |
|  | 20 | If repeated inquiries, whether score of each inquiry was reported? |
|  | 21 | If multiple evaluators, is consistency evaluated? |
