# Supplementary material for: Performance of ChatGPT Across Different Versions in Medical Licensing Examinations Worldwide: Systematic Review and Meta-Analysis
Source: J Med Internet Res. 2024 Jul 25;26:e60807. doi: 10.2196/60807 (PMC11310649; doi:10.2196/60807)
Supplement: Multimedia Appendix 4 [file jmir_v26i1e60807_app4.pptx]

## Slide 1
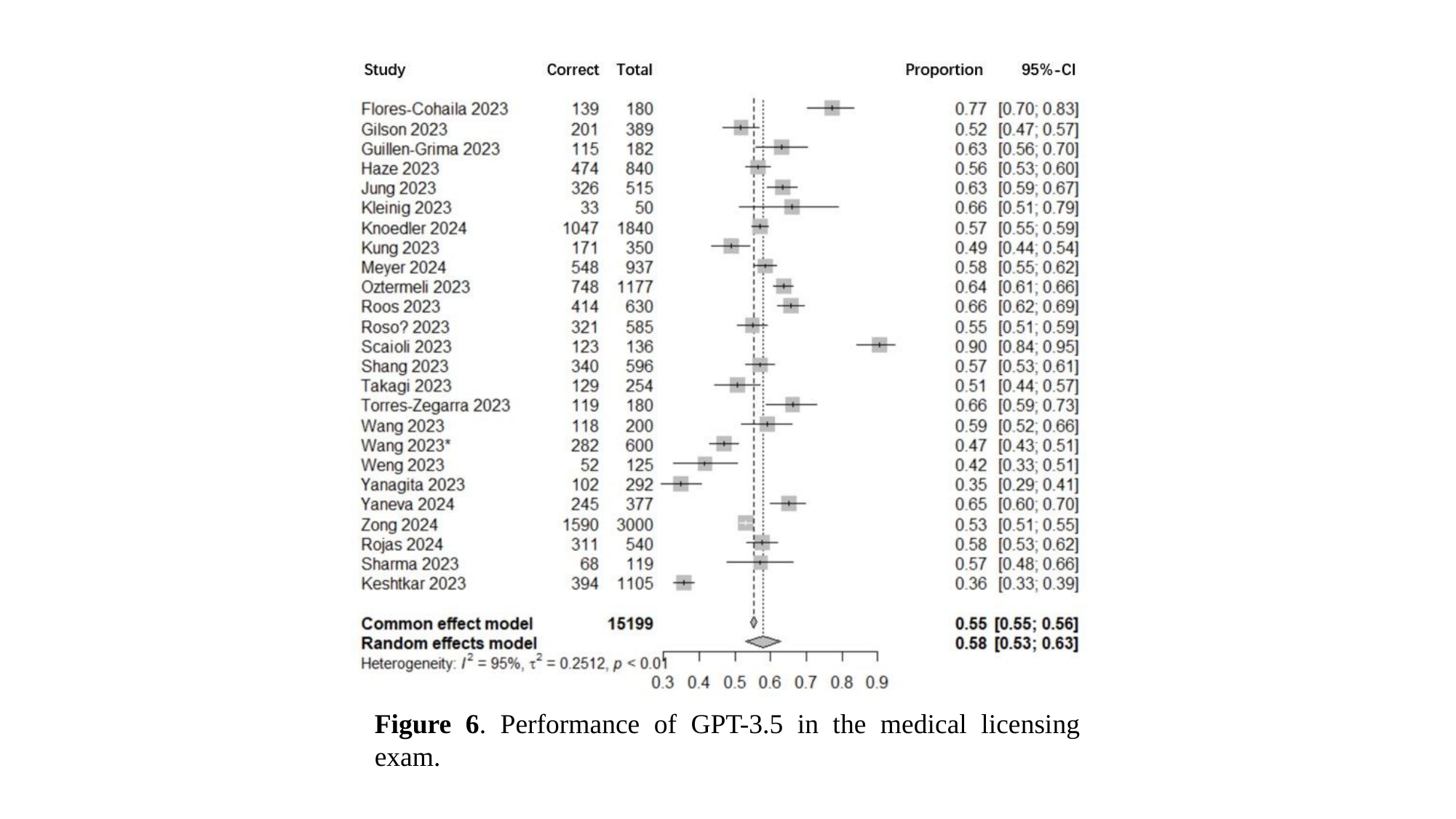

Figure 6. Performance of GPT-3.5 in the medical licensing exam.

## Slide 2
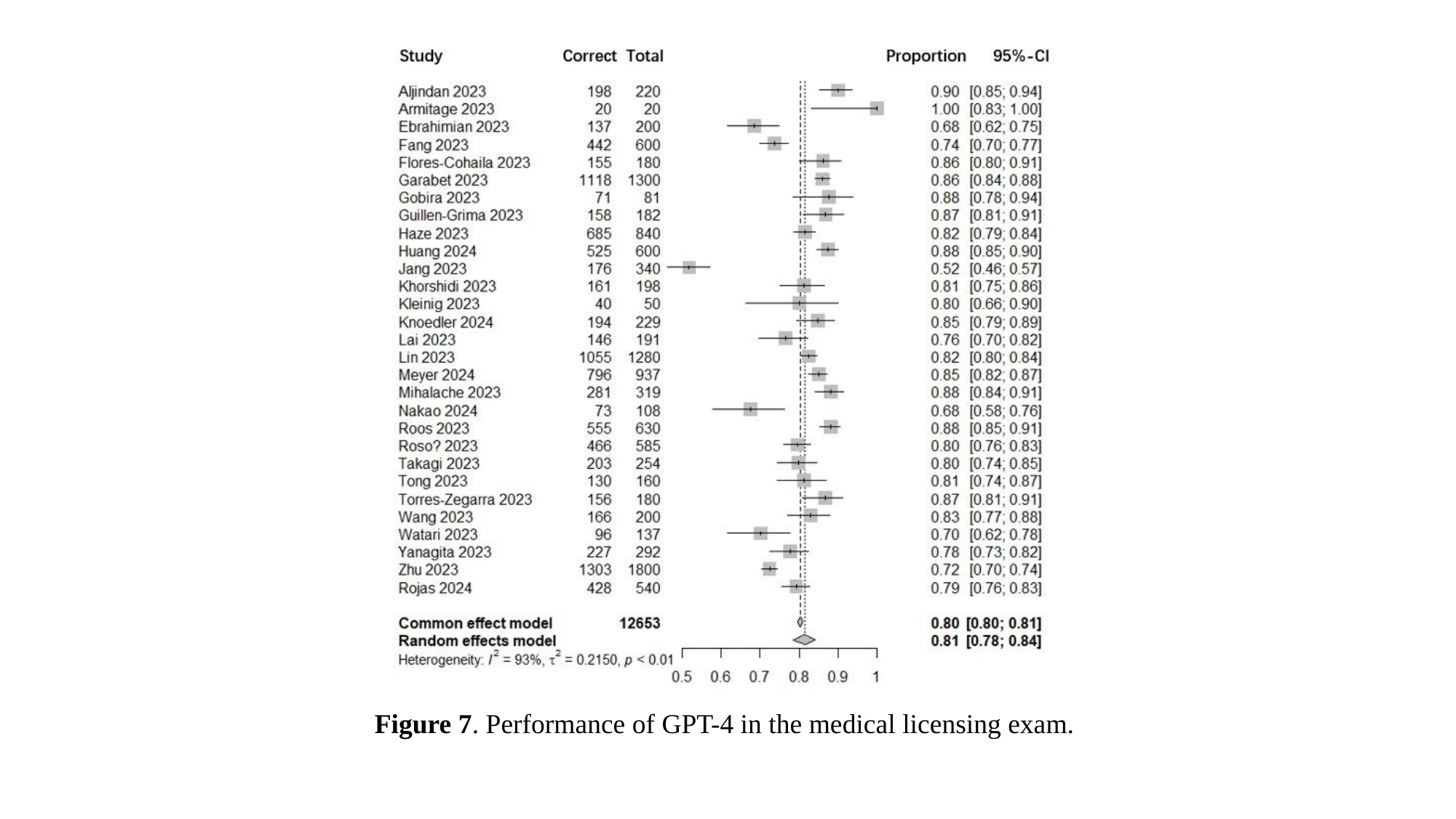

Figure 7. Performance of GPT-4 in the medical licensing exam.

## Slide 3
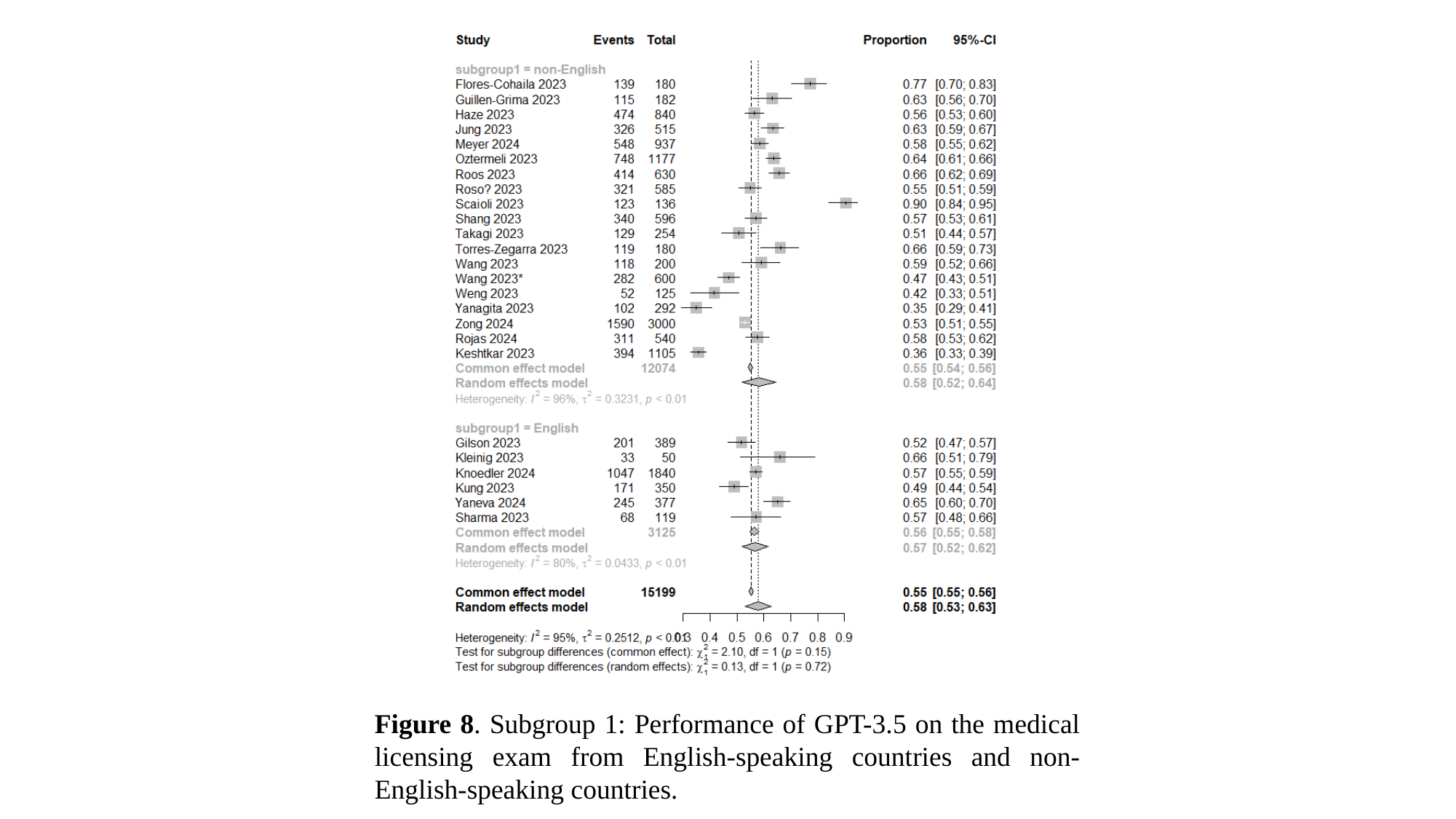

Figure 8. Subgroup 1: Performance of GPT-3.5 on the medical licensing exam from English-speaking countries and non-English-speaking countries.

## Slide 4
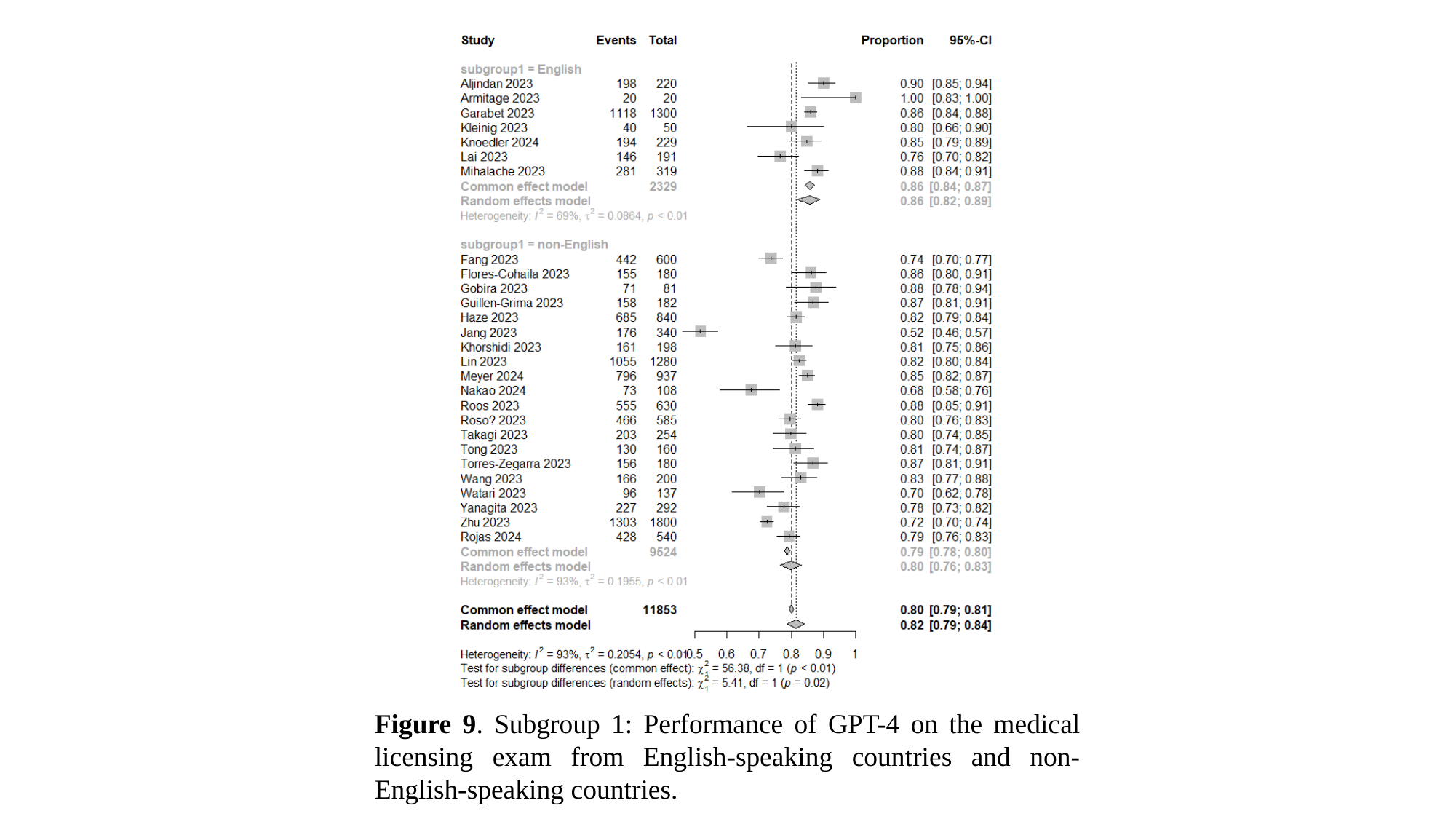

Figure 9. Subgroup 1: Performance of GPT-4 on the medical licensing exam from English-speaking countries and non-English-speaking countries.

## Slide 5
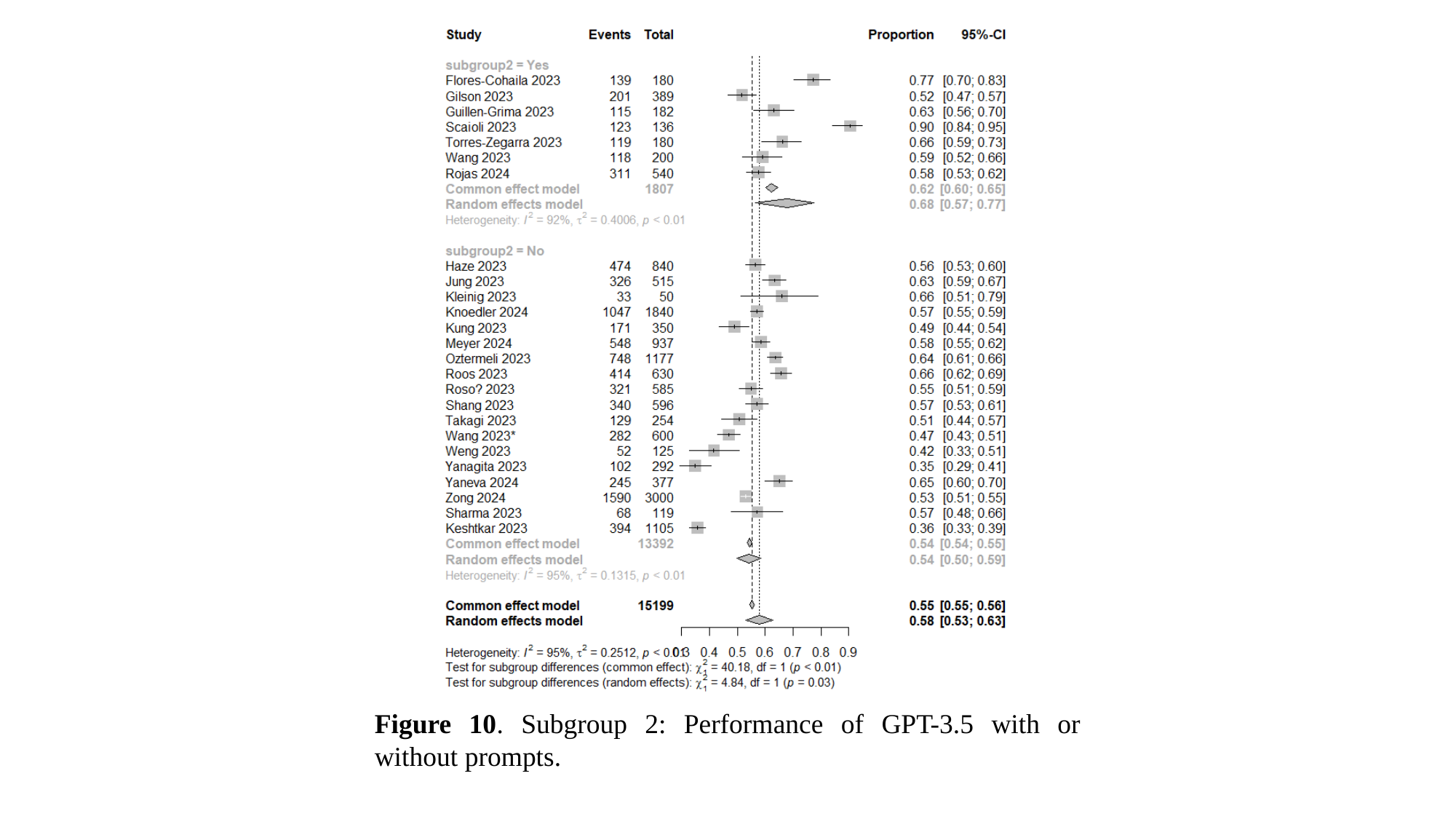

Figure 10. Subgroup 2: Performance of GPT-3.5 with or without prompts.

## Slide 6
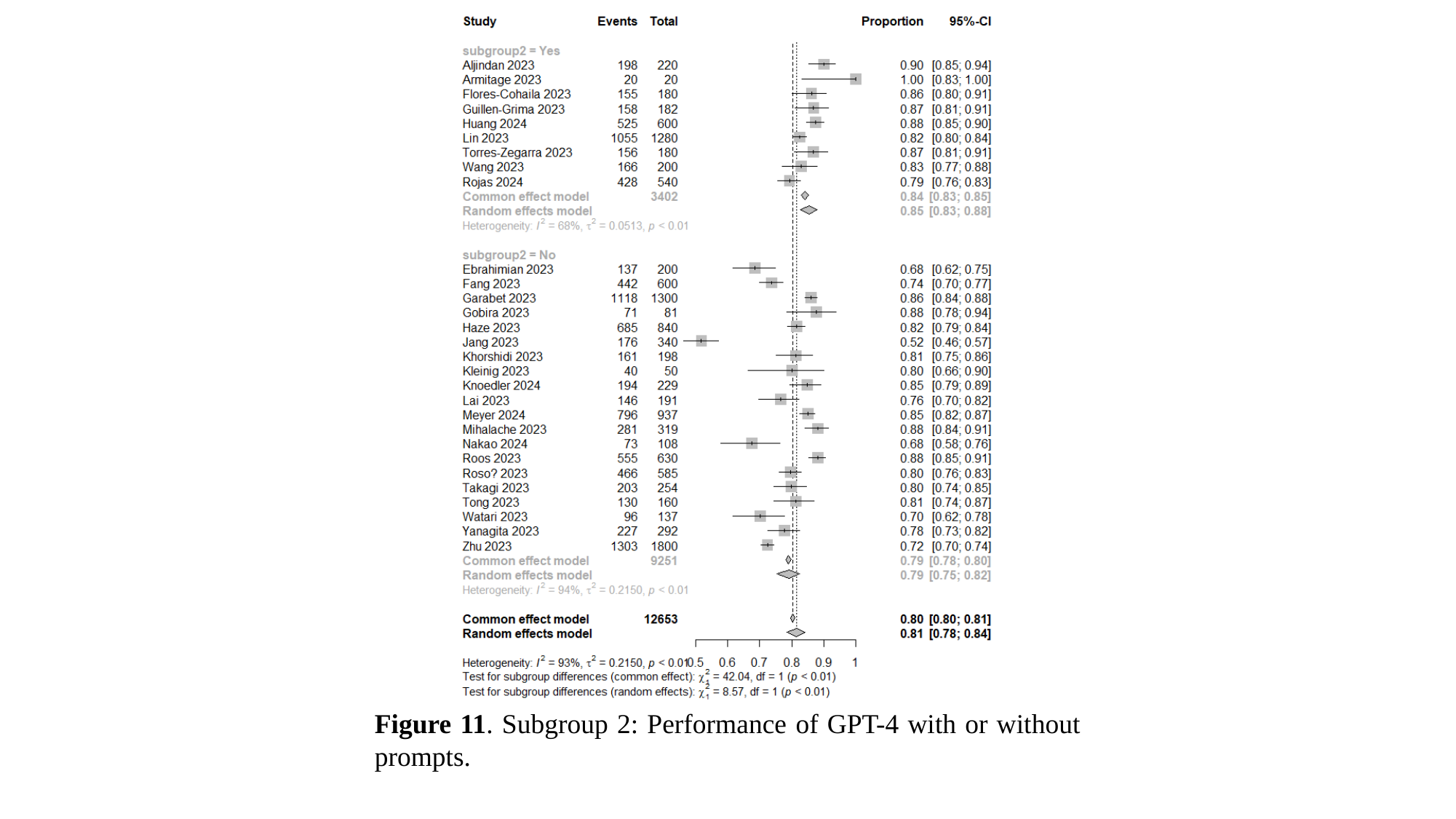

Figure 11. Subgroup 2: Performance of GPT-4 with or without prompts.

## Slide 7
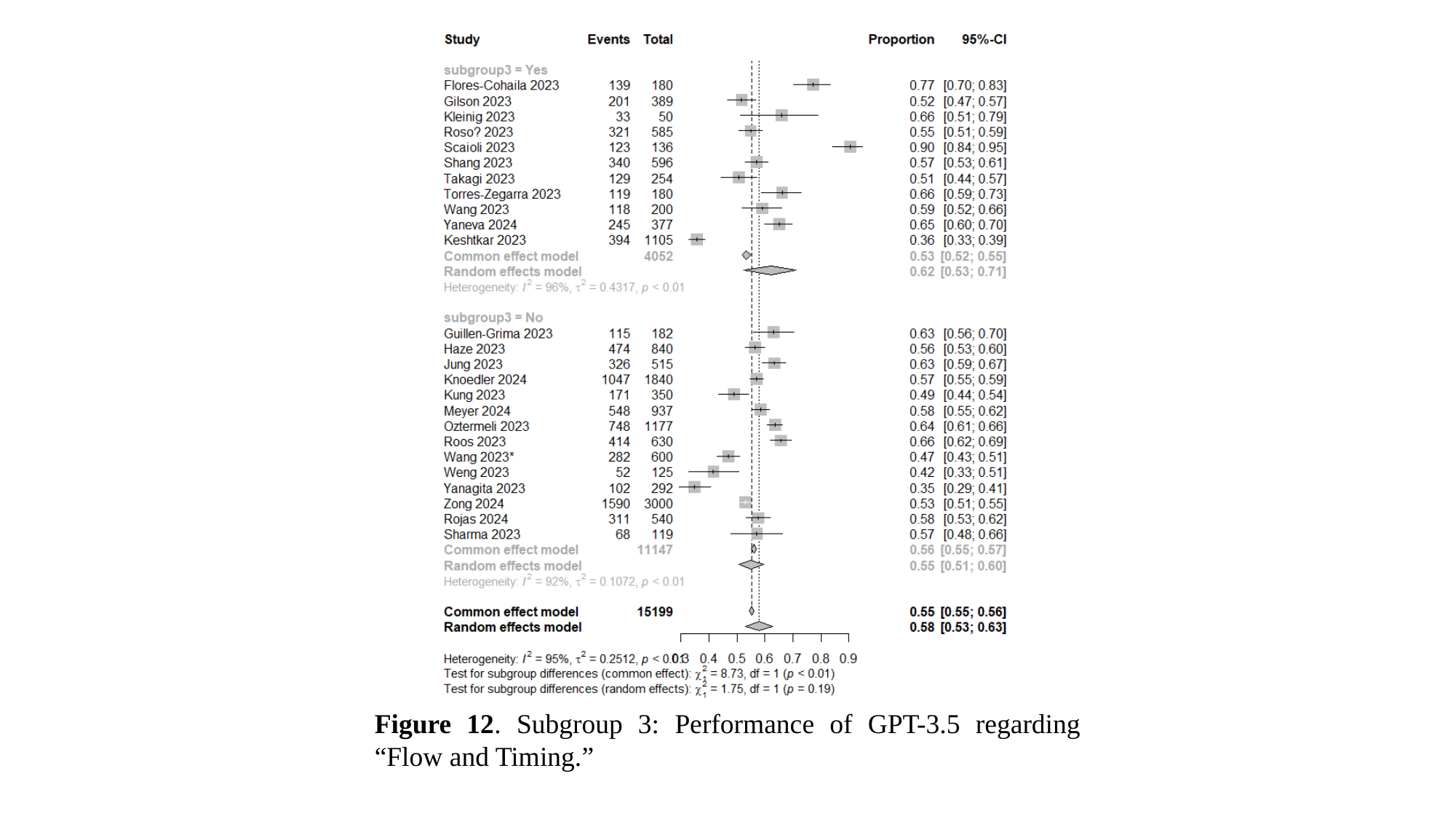

Figure 12. Subgroup 3: Performance of GPT-3.5 regarding “Flow and Timing.”

## Slide 8
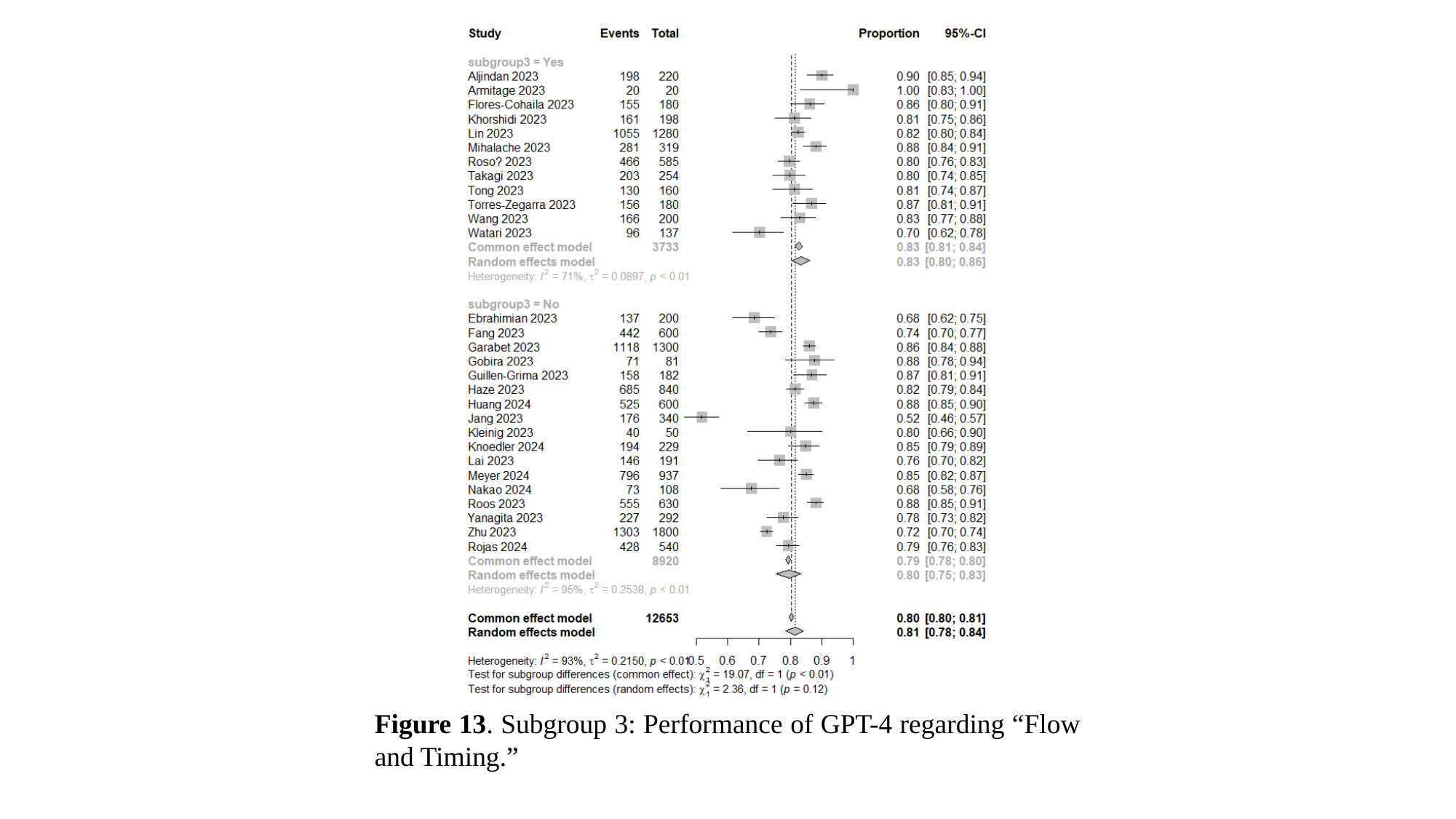

Figure 13. Subgroup 3: Performance of GPT-4 regarding “Flow and Timing.”

## Slide 9
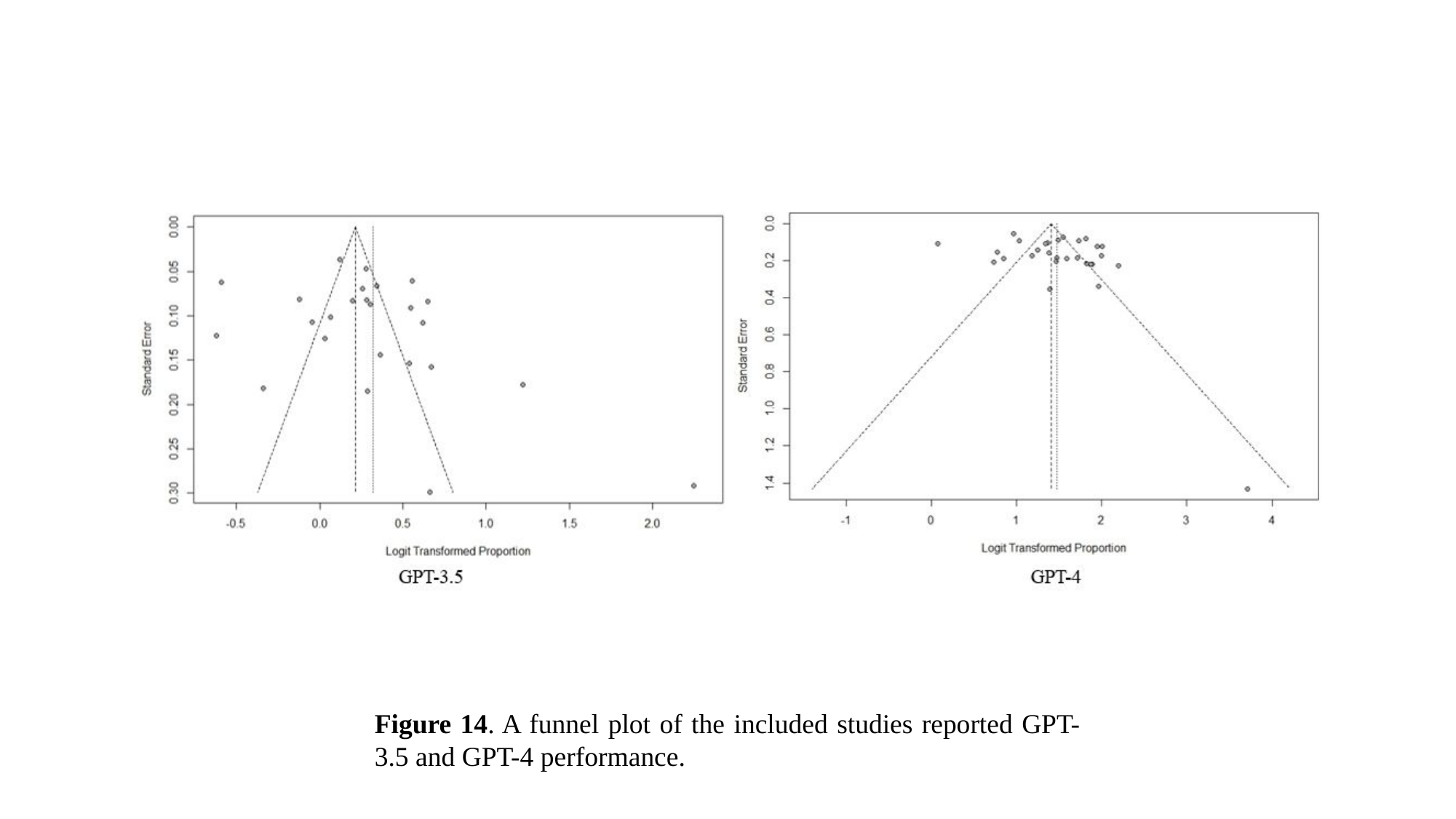

Figure 14. A funnel plot of the included studies reported GPT-3.5 and GPT-4 performance.

## Slide 10
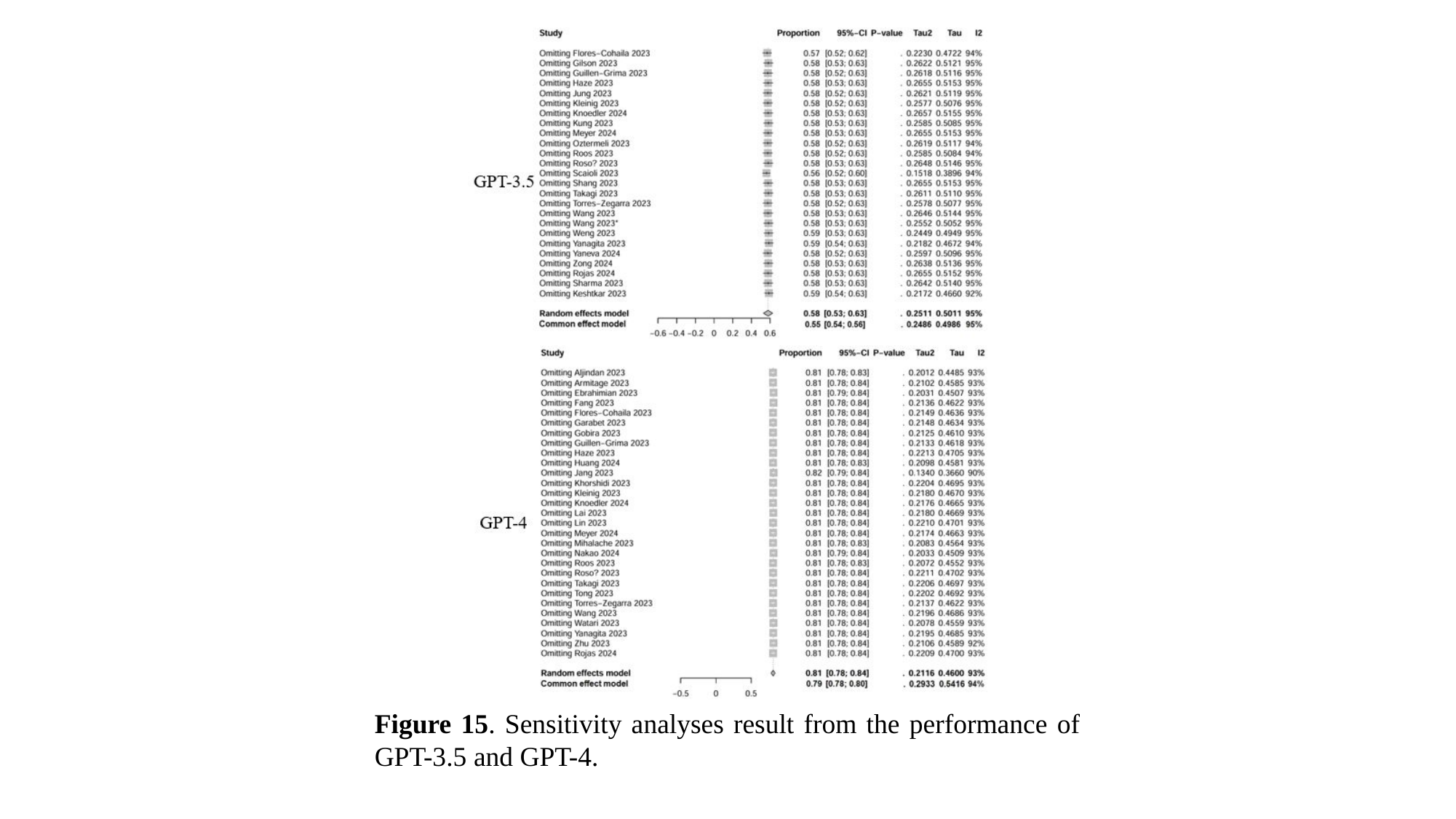

Figure 15. Sensitivity analyses result from the performance of GPT-3.5 and GPT-4.
